# Post-normal: Crisis and the End of the Ordinary

**DOI:** 10.1177/1329878X20958151

**Published:** 2020-11

**Authors:** Elizabeth Stephens

**Affiliations:** The University of Queensland, Australia

**Keywords:** 2016 election, 2019 bushfires, normality, pandemic, temporality

## Abstract

This article examines the ubiquity of discussion about the normal in media commentary on the coronavirus pandemic. Almost all media commentary on the pandemic references a hope that things will soon, or at least eventually, ‘return to normal’. Yet what is meant by the normal is rarely the subject of explicit description; rather, its meaning is almost always taken for granted. This article examines the centrality of the concept of the normal to media commentary on the pandemic, showing that its significance is affective as much as semantic.

Of all the unexpected things the arrival of the plague brought with it, the distortion of time was one of the strangest. At the beginning of 2020, as across the world, cities and then entire countries moved into lockdown, and hundreds of millions of people found themselves suddenly and unexpectedly isolating in their homes, one of the most remarked open experiences of the pandemic was the widespread sense that temporality itself had warped. Pandemic time seemed both radically accelerated and interminably slowed down: ‘Something has happened to time’, as Arielle Pardes (2020) noted in an article on ‘coronatime’ for *Wired*:

The virus has created its own clock, and in coronatime, there is less demarcation between a day and a week, a weekday and a weekend, the morning and night, the present and the recent past. The days blend together, the months lurch ahead.

This distortion of time has been the subject of endless pandemic memes and jokes. Some of these play with tense, like the mock complaint: ‘Next week has been exhausting!’ Others play with the disruption of temporality: calendars represented by celebrity portraits that age decades in months; or which, conversely, are presented by an identical static pose month after month after month. The instant and commonplace uptake of the terms Before Times, Plague Times and After Times is a further reflection of the radicality of this temporal disruption, and the widespread sense of an epochal transformation it has brought with it.

In many ways, however, this widespread public perception of a profound disruption in temporality provides a cultural and discursive focal point for what is a much more generalised sense of strangeness and disorientation caused by the upheaval of the pandemic, and the deep sense of cultural unmooring to which it has given rise. As so many across the world found their lives knocked off-kilter, forced to adjust to new lives as full-time internauts pinwheeling through a virtual deep space of online meetings and classes, it became clear that the pandemic had produced a crisis not just in public health but in our very capacity to make sense of the world. The pandemic, then, must be approached as an experience as well as an event. This experience is one characterised for many by a pervasive and widely shared sense of strangeness, a much-remarked awareness of its lack of historical precedence, which has thrown not just ordinary life but the very idea of the ordinary into deep crisis. In this article, I want to examine how this sense of weirdness and disorientation caused by the pandemic has given rise to a discursive explosion in both traditional and social media whose incidence and significance remain largely unrecognised, and which focuses on the return and the future of the ‘normal’.

The word ‘normal’ occupies a place so ubiquitous in media discussions about the pandemic that it would be hard to overstate its discursive dominance. Headlines and articles speculating on whether things will or should go back to normal after the pandemic number in their hundreds of thousands (as of July 2020). As Siddhartha Mukherjee (2020) noted recently in the *New Yorker*, the future of the normal is the central question of the pandemic: ‘Everyone now asks: When will things get back to normal?’ Curiously, however, given this discursive centrality, the word normal itself is almost never described or defined in these articles. Instead, its meaning is simply taken for granted, as though everyone is so familiar with this word that its meaning requires no further elaboration. As a result, one of the most dominant discursive and conceptual frameworks through which the pandemic has been, and continues to be, discussed and understood remains remarkably vague and outside the sphere of public scrutiny. Given this, there is a critical value in turning to examine the specificity of this term and to ask what it exactly means in the various contexts in which it is being used. The aim of this article is thus to examine and make visible the work the word ‘normal’ is doing in media discussion about the pandemic, drawing on both traditional and social media examples. There is a strong link between the current sense of historical and temporal crises and the discursive proliferation of the normal in pandemic media commentary, and I seek to show here, which can be best understood by recognising the particular context from which this current usage has emerged.

## This is not normal: political crisis and the US 2016 presidential election

The current discursive dominance of the normal in media commentary about the pandemic has its roots in an earlier recent moment of crisis: that produced by the previous US Presidential Election in 2016. At a Democratic rally held during the final weeks of the 2016 US presidential election campaign, Michelle Obama responded to the then-recently publicised recordings of Trump boasting about sexually harassing the women with whom he had come into professional contact with a combination of outrage and dazed disbelief that has become so familiar to discussions about the current Presidency: ‘I can’t believe that I’m saying that a candidate for president of the United States has bragged about sexually assaulting women’, she told the crowd. ‘Too many are treating this as just another day’s headline. As if our outrage is overblown or unwarranted. As if this is normal. Just politics as usual’. She concluded, emphatically, ‘This is *not* normal. This is *not* politics as usual. This is disgraceful, it is intolerable’. This angry rejection of Trumpian values, and the insistence that his candidacy represented an unprecedented break from the usual state of affairs in American politics, epitomised reactions to the 2016 election campaign in the left-leaning media publications and platforms (Stephens 2019). It has continued to characterise reactions by the political left to the current US President in the years since. In the run-up to the 2016 election, headlines and political analysis focused on whether the current state of affairs in American politics could be described as normal, not normal or evidence of a dangerous new normal: ‘2016 Isn’t Normal’, the *US News* website declared (Fenn, 2016); ‘Don’t Let Donald Trump Become the New Normal’, the *Guardian* urged (Thrasher, 2016); ‘Welcome to Washington’s New Normal: One Trump Drama After Another’, *The Washington Post* warned (Rucker and Fisher, 2016). Headlines such as these dominated the election coverage in the traditional media, while the hashtag #notnormal proliferated in social media commentary.

While the word ‘normal’ here is again rarely defined or described, despite its evident centrality to the public discussion that took place in traditional and social media outlets at this time, what was at stake here, as Michelle Obama’s remarks above indicated, was the question of what was, or should be, ‘acceptable’ or ‘tolerable’. This understanding of the normal, and especially its linking to the ‘acceptable’, represented something new in 2016. The semantic novelty of the normal, as it was used in public and media commentary about the 2016 election, produced a significant discursive legacy, introducing into the American vernacular what was then a new word: ‘normalisation’. Writers for publications including the *Guardian, New Yorker* and the *Boston Globe* all identified ‘normalisation’ as their word of the year at the end of 2016, as did the Merriam-Webster dictionary website (2016): ‘It will sometimes happen that a word suddenly appears everywhere. In the wake of the 2016 presidential election, two such words are currently in the ether: the verb *normalise* and its related noun, *normalisation*’.^1^ While the word ‘normalisation’ was of course not new in 2016, as Mark Peters argued in the *Boston Globe*, the sudden and emphatic entrance of the word ‘normalisation’ into popular discourse at the end of 2016 represented a new and distinct meaning of the word. Where previously the ‘normalisation’ had been primarily used to refer to the process of making something more normal, in 2016, it was overwhelmingly used to refer to the process of making the abnormal seem widely acceptable: ‘These days, people are using *normalize* to mean “shift our perception of normal to include a thing previously seen as abnormal”’, Peters (2016) explained, ‘rather than “change an abnormal thing to make it conform to a norm”’.^2^ Emily Dreyfuss neatly summed up the conceptual dominance of the normal and normalisation in the election coverage in an article for *Wired* magazine, entitled ‘The Normalization of “Normalize” Is a Sign of the New Normal’, which noted that ‘Americans are using [the word] in a different way than they normally do. The country is normalizing a new use of “normalize”’ (Dreyfuss, 2016). ‘Normalisation’ as used in the context of the 2016 US election thus provided a new word with which to name what was widely perceived as new dynamic, one that was causing a perceived status quo to undergo a rapid, radical and unwelcome change.

The identification of Trump and the Trump presidency as ‘not normal’ and as something that should not be normalised provided a way to identify, and denounce, that presidency as ‘unacceptable’ or ‘intolerable’. The word ‘normal’ here thus named a state of affairs that was perceived to be under threat. As a result, the 2016 US election provoked a sustained and often heated debate about the state of American politics, in which the word ‘normal’ served as a flashpoint, the name of a cultural space that was understood to be endangered and under attack. What is particularly striking about the use of ‘normal’ in this context is the extent to which it is attributed with a positive value, identified as something to be protected and safeguarded: ‘We’re quite protective over the concept of normal’, as Jessica Brown noted of the election on the BBC website. ‘After a big life event, all we want is to go back to normal. It’s our default, our comfort zone’ (Brown, 2017). The normal as it is used here refers to something comforting and familiar; an ordinary time that preceded a current state of crisis and chaos. However, the ‘comfort zone’ named here, it should be recognised, is that of the political left. Conservative voices and those to the hard right were not to be found writing in defence of the normal during the election campaign or its aftermath, nor did they apparently perceive the normal as something under attack.

One of the most striking things about this defence of the normal is that it is advanced largely by those on the political left, and that it does so immediately following decades of critique of this term by scholars who largely share this political affiliation. For until very recently, discussions about the normal, especially by those on the political left, focused almost exclusively on its negative meanings and effects. In contemporary critical and cultural theory, for instance, the normal has been the subject of sustained and detailed critique in recent years. Particularly in feminist and queer theory, as well as in disability and race studies, the terms ‘normal’ and ‘normativity’ have been widely critiqued as practices of enforced conformity and standardisation, which naturalise existing and harmful systems of privilege.^3^ In order to understand what this has to tell us about the status and significance of the normal itself in the present day, it is useful to contrast the calls to resist the normalisation of a Trump presidency cited above with the critiques of normativity articulated in these fields of contemporary critical theory. Both normalisation, as used in the media commentary above, and normativity, as used in contemporary critical theory, are understood as negative dynamics that impede or damage cultural diversity and inclusiveness. At the same time, however, critiques of normalisation and normativity are predicated on strikingly different understandings of the normal itself. Calls to resist normalisation in the wake of the 2016 election were made in defence of the normal: normalisation must be resisted, it was claimed, in order to prevent damage being done to the normal. That is, the normal was understood to be harmed by normalisation, whereas normativity often understands norms themselves as harmful to those upon whom they are imposed (Stephens 2014).

If, after decades of critique and announcements of its cultural redundancy, the word ‘normal’ can once again surge into popular use as a culturally central and vital concept, and if it can be so readily adopted by those on the political left as a cultural state that needs to be, and should be, protected against the rise of hard-right and authoritarian governments, then we are dealing with a word whose meaning is unusually volatile and metamorphic. In current usage, the idea of the normal and the not normal provide a conceptual spectrum within which to register a sense of affective and cognitive shock. To lose one’s sense of the normal, to feel the normal dissipating or transforming around one, is to feel as though the world itself has gone mad. As David Remnick, editor of the *New Yorker*, marvelled to CNN immediately after the election: ‘When I hear [Trump] described as not sexist, not racist, not playing on white fears, not arousing hate, when he’s described in a kind of normalised way [. . .] I think I’m hallucinating’ (CNN, 15 November 2016). In the *New York Times Magazine*, essayist and critic Teju Cole compared the aftermath of the 2016 election to the Ionesco play *Rhinoceros*, in which a sighting of a rhinoceros elicits first outrage and disbelief among the townsfolk, then acceptance and, eventually, an epidemic of ‘rhinoceritis’ as one by one all the characters transform into rhinoceroses. Just as Ionesco’s play, written in 1959, was widely taken as a commentary on the upsurge of fascism prior to the Second World War, so must contemporary Americans resist the normalisation of a new and dangerous authoritarianism embodied in the monstrous figure of Trump, Cole (2016) argued.

However, it is important to recognise that not all commentators agreed the 2016 election campaign *did* represent a break with the normal. We see this in the (much smaller) pool of commentary that questioned the perception that the Trump candidacy signalled a significant break or rupture with normal American political practice or social values, noting that the widespread disbelief in the face of Trump’s stated views – regarding women, people of colour or people with disabilities – was mostly confined to a White, progressive middle class. As Hua Hsu (2016) reminded readers of *The New Yorker*: ‘Racism, sexism, and the other hatreds and phobias lately on display . . . have always been normal – for some of us’. *The Washington Post*, too, acknowledged that while ‘Donald Trump’s election as president startled many Americans’, the widespread perception that the ‘illiberal values and policy positions’ espoused during his campaign were ‘far outside of the United States’ political traditions’ was incorrect: ‘in many ways, Trump represents a return to the historical norm’ and its ‘set of commitments to hierarchies of race, nationality and religion, among others’ (Klinker and Smith, *The Washington Post*, 17 November 2016). The perception that something new and dangerous was happening to the country, the fear that this represented an unprecedented break from historical norms or social reality, was thus largely confined to a fairly privileged group. As Courtney Parker West (2016) wrote in the *Huffington Post*, the shocked reactions of White liberal Americans to the election campaign were itself a manifestation of White privilege:

spare me the advertisement of just how shocking it all is [. . .] because some of us – *my* little black and Indigenous ass – [. . .] are not aghast that presidents who say bogus shit dance their way into office. We have seen this before. [. . .] I am devastated, but no, I am not shocked.

These tensions remain in stark evidence in 2020, as the ongoing pandemic lockdowns have been intersected by the global Black Lives Matter and Indigenous Lives Matter protests taking place at the time of writing, and which constitute an important backdrop to the debate about the normal taking place in the context of media writing on the pandemic. The (re)emergence of the normal as discursively central to US political discourse in 2016, and the context of widespread cultural crisis in which this took place, can be directly traced into contemporary media commentary about the pandemic during 2020. Before doing so, however, it is worth pausing at an intermediary moment, which arose during the Australian bushfires that devastated the country at the end of 2019.

## Welcome to the new normal: climate crisis and the 2019 Australian bushfires

In July 2019, a series of bushfires began in Australia that would burn uncontrolled for the next 9 months, impacting every state in the country, and causing widespread ecological devastation. In the months to come, the states of New South Wales, Victoria and the Australian Capital Territory would all declare states of emergency. The summer of 2019–2020 became the most severe fire season on record. By its end, 34 people had died, millions of hectares of land had been incinerated and an estimated billion animals, including entire populations of endangered species, had perished with it. Regional areas were impacted most severely, with fires obliterating ancient sacred sites and destroying regional townships. However, coastal cities did not escape the effects. For months, the skies over the eastern coast of Australia were an eerie sunset red all day, an apocalyptically red sky from a science fiction movie, heralding a bleak future to come. The air was unbreathable, with air pollution levels 10 times over the officially ‘hazardous’ levels. In a foreshadowing of a future Australians did not yet know was to come, for many it became necessary to wear masks to go outdoors, while the medically vulnerable were forced to stay indoors. The last of the bushfires was extinguished in March 2020. This milestone went largely unremarked, however, as the country was then heading rapidly towards a full lockdown due to the pandemic just taking hold in the country.

Although extreme bushfire seasons have been common in Australia since – and because of – White settlement, and its forced cessation of Indigenous cultural burning practices, the summer of 2019 and 2020 was nonetheless alarmingly far from normal, as it was widely reported. Bushfires that tore through rainforest – rainforest that had never burned before – fire fronts that joined together across multiple state lines to produce megafires and the extreme air pollution that resulted were all recognised as something new; dangerous signs of a changing climate and another environmental tipping point. The phrase ‘climate grief’ entered the popular Australian vocabulary, as Indigenous communities were forced to deal with yet another devastating effect of White settler culture: ‘It’s a particular grief, to lose forever what connects you to a place in the landscape’, Lorena Allam lamented in the *Guardian*:

Our ancestors felt it, our elders felt it, and now we are feeling it all over again as we watch how the mistreatment and neglect of our land and waters for generations, and the pig-headed foolishness of coal-obsessed climate change denialists turn everything and everyone to ash. (Allam, 2020)

As a result of their extent and impact, the 2019 bushfires provoked an international debate about the rate and scale of climate change and of the role of government policy in mitigating this. In the midst of this debate, the Australian Prime Minister Scott Morrison declared, in a widely reported announcement, that such fires were now the ‘new normal’ to which Australia would just have to adapt: ‘We have to prepare for the new normal’, he said, arguing that the government response needed to focus on ‘resilience and mitigation’ rather than the wider issue of climate change and environmental policy or regulation (see, for instance, Speers, 2020). Morrison’s statements were widely reported internationally, as well as within Australia.^4^ While the phrase ‘the new normal’ is one that was in widespread popular use prior to the 2019 bushfire season, its meaning in this context marked a pivotal moment in the coupling of looming crises with a re-evaluation of the normal. The ‘new’ part of this phrase referred to a set of conditions generally understood to be unpleasant at best and catastrophic at worst: increased fires and an increased level of environmental devastation in consequence. The ‘normal’ here referred to a continuation of a status quo in which Australia continued to enable and facilitate its fossil fuels industry at the expense of long-term economic and environmental sustainability. By announcing the fires as ‘normal’, albeit a new and disagreeable normal, government was widely understood to be rejecting any need for government action or intervention on the wider issue of climate change.^5^ The insistence that Australia would have to ‘adapt’ to climate crisis was thus simultaneously an assertion that it was possible to adapt to it. The severity and unpredictability of these fires was thus redefined as part of a new status quo requiring change and resilience on the part of the populace, rather than symptomatic of an environmental crisis that would need to be addressed in policy. This use of the phrase ‘new normal’ resonates with its use in the context of the 2016 US presidential election, as examined above: here, the phrase became a subject of debate because the expectation that such fires were something to which the country or populace could adapt was widely taken to be an unacceptable or even intolerable response.

This can be seen in the way that, rather than reassuring the public with appeals to normality, Morrison’s description of the fires as Australia’s ‘new normal’ conversely served to provoke anger and anxiety about the scale and impact of climate change and concern about the government’s denialism and intractability in addressing this. In the ensuing debate, echoes of the new meaning of ‘normalisation’ that emerged during the 2016 election could be heard again: what had formerly been recognised as extreme was now becoming common. The ‘new normal,’ for which Australia must prepare itself, was one in which the limits of the acceptable and endurable had been recalibrated: ‘the recent seasons have firefighters rethinking what should be considered normal’, noted the ABC. Similarly, QFES acting deputy commissioner, Neil Gallant argued: ‘We’ve got to be prepared for a different fire season, a different range of climate extremes. We’d be not doing our duty if we didn’t at least consider that’s now the new norm’ (Walker, 2020). The ‘new normal’, as it was used in this context, was thus largely understood to refer to a formative status quo, one whose novelty was primarily experienced as unpleasant. The ‘new normal’ here named a fault-line; the debated territory of adaptability. It is this understanding of the normal, as a contested zone of adaptation and adaptability, that would come to underlie much of the media commentary on the 2020 pandemic.

## Weird times: the crisis of the normal and the 2020 coronavirus pandemic

By the time the novel coronavirus epidemic became a pandemic in March 2020, then, whatever passed for the normal in popular discourse and culture had been in serious trouble for some time: ‘As we grapple with uncertainty and upheaval, it’s clear that our old “normal” will never be recovered’, reflect Milne, Hendriks and Mahanty in an article in *The Conversation*, reflecting on the difficult Australian summer that was then giving way to the tumultuous year of the pandemic. In consequence, while discussion about whether we can or should ‘return to normal’ remains ubiquitous in media commentary on the pandemic, and while the desire to ‘feel normal’ again continues to characterise self-representations of the pandemic experience, in recent months, a mounting critique of the normal has been steadily gaining traction: ‘“Normal” life failed us’, John Harris declared in the *Guardian*. ‘On a bad day, our national nightmare now appears so deep and complex as to feel not just depressing, but insurmountable. Any return to the “normal” that has so horrifically failed us is unthinkable’ (Harris, 2020). For Paul Carr (2020), any attempt to ‘return to normal’ would be ‘inhumane’. Increasingly, it is precisely what we once accepted as normal that has come to be identified as the very source of the problems that have brought us to this point of calamity. As Ed Yong (2020a) summarised in a recent and widely shared article for the *Atlantic*, ‘How the Pandemic Defeated America’:

The U.S. cannot prepare for these inevitable crises if it returns to normal, as many of its people ache to do. Normal led to this. Normal was a world ever more prone to a pandemic but ever less ready for one. To avert another catastrophe, the U.S. needs to grapple with all the ways normal failed us.

Accordingly, a growing number of voices have recently argued that, instead of seeking to return to the normal, to an ordinary world familiar but toxic, we should seize this moment of crisis to do away with it entirely: ‘crisis leads to a fork in the road’, as Jack Halberstam argues. ‘One way rights the balance and leads back to “normal life”, the other moves in the opposite direction and leads elsewhere with outcomes that are unknown’ (Halberstam, 2020). The upheaval caused by the pandemic provides the opportunity to reimagine the world, to step into the unknown future by dismantling the systems of exploitation and extractive capitalism that have brought us to this moment. In one of the most widely shared early reflections on the pandemic, the writer Arundhati Roy (2020) captured the sense of the momentousness and promise of this moment for many:

What is this thing that has happened to us? It’s a virus, yes. In and of itself it holds no moral brief. But it is definitely more than a virus. [. . .] Whatever it is, coronavirus has made the mighty kneel and brought the world to a halt like nothing else could. Our minds are still racing back and forth, longing for a return to ‘normality’, trying to stitch our future to our past and refusing to acknowledge the rupture. But the rupture exists. And in the midst of this terrible despair, it offers us a chance to rethink the doomsday machine we have built for ourselves. Nothing could be worse than a return to normality. Historically, pandemics have forced humans to break with the past and imagine their world anew. This one is no different. It is a portal, a gateway between one world and the next. We can choose to walk through it, dragging the carcasses of our prejudice and hatred, our avarice, our data banks and dead ideas, our dead rivers and smoky skies behind us. Or we can walk through lightly, with little luggage, ready to imagine another world. And ready to fight for it.

At the end of a period that has seen a rapid move to the hard right politically in Australia as well as in the United States (along with many other regions of the world), while witnessing the increasingly extreme effects of climate change and ecosystem destruction, the normal has come to name a cadaverous status quo, a wasteland of dead ecosystems and extractive capitalism. Here, the positivity that accrued to the term ‘normal’ during the turmoil of the Trump election has disappeared. The normal here is not to be defended or protected; it is instead the source of harm. Thus, while references to wanting things to get back to normal remain routine to the point of ubiquity in popular media commentary on the pandemic, where the word is subject to explicit discussion, it is increasingly understood in negative terms: as a status quo that is no longer sustainable or endurable.

It may seem, given this, that the concept of the normal is unlikely to survive the upheavals of the pandemic; that, as a word, it is now likely to fall into redundancy or disuse. However, it should be remembered that the normal, as a concept, has always been in trouble; indeed, as we have seen above, it most commonly and insistently appears not where the status quo is most stable or secure but on the contrary where it is most troubled and perceived to be in crisis. More than 20 years after the publication of Michael Warner’s (1999) landmark book of that title, *The Trouble with Normal*, then, the idea of the normal remains as problematic as ever, yet also as resilient. Indeed, as we have seen above, it is often immediately after the normal has been most subject to sustained critique and pronounced discursively dead that it surges most forcefully in its frequency of use and cultural centrality. This can be seen in its abrupt readoption in criticism of the Trump election campaign and presidency, starting in 2016, and it can also be seen in current media commentary on the pandemic. That the ‘normal’ can be so widely used in both positive and negative ways perfectly exemplifies its semantic capaciousness and changeability.

As we have seen above, the ‘normal’ is a term that tends to come to the fore, and whose usage dramatically spikes, during those moments in which its meaning is most contested, and when it is most perceived to be in crisis. In our long history of the normal, *Normality: A Critical Genealogy*, (Cryle and Stephens 2017), Peter Cryle and I discovered this to be a consistent characteristic of the normal across the modern period. It is a term that has long been subject to periodic denunciations and a concept that has often been assumed to be culturally moribund. Yet, it continues to return to a position of cultural centrality and discursive dominance time after time. There is an especially strong recourse to the idea of the normal in times of crisis. The normal, as the cases examined above have shown, provides a conceptual framework in which such crises can be identified and examined. Accordingly, this article has sought not to evaluate or adjudicate the merits of the normal but to examine its use and meaning. It is designed neither to celebrate nor condemn, but to attend to the conditions of its cultural persistence and conceptual resilience. What is at stake in this is recognition of the unspoken terms and assumptions embedded in these debates about the crises caused by the pandemic, including the uneven racial distribution and effects highlighted during the ongoing Black Lives Matter and Indigenous Lives Matter marches. The sense of crisis and disruption felt by so many in the present moment, and which is seen to mark such a definitive break with the past, is not a new experience for many. Yet the normal remains a weathervane for many, and naming its absence is a way of marking the limits of what one can cope with or adjust to. The ‘normal’ names an ordinary state that has been superseded by a constant state of emergency and disaster. Especially during moments of crisis, the ‘normal’ names a state of comforting familiarity to which many become more attuned when faced with its absence. In such a context, recognising the centrality of this term, and attending to the specific dynamics it names in each instance, can help us unpack the terms in which the current crises caused by the pandemic are being understood and the extent to which that conceptual infrastructure might come to shape the as yet uncertain futures to which it will give rise.

## Notes

1. Merriam-Webster Website (2016), ‘The New “Normalise”: Is The Meaning of “Normalization” Changing?’ November. See also: Peter Bradshaw (2016); Emily Dreyfuss (2016); James Harbeck (2016); Hua Hsu (2016); and Mark Peters (2016).

2. Prior to 2016, as the Merriam-Webster website noted, the word normalise was primarily used in technical and scientific writing, or in political theory, to describe ‘a return to a state considered normal. Later, it was used to describe the act of making something variable conform to a standard’. The word was used this way in international relations and political theory, to refer to the ‘normalisation’ of relations between countries, and in medicine, to refer to the ‘normalisation’ of biological function. It was also used in a quantitative sense to refer to ‘data normalisation’, in which redundancies and anomalies are removed from a database. But in the days immediately preceding the election, and then in the 2 weeks immediately after it, the word began to be used in a new way: ‘Recently, we’ve seen it used to describe a change in what’s considered standard. In this new “normalization”, the standards change to make something considered an outlier “normal” – not the other way around’ (‘The New “Normalize”: Is the meaning of “normalization” changing?’).

3. Representative texts include Dean Spade’s (2011) *Normal Life: Administrative Violence, Critical Trans Politics, and the Limits of Law*; Julian Carter’s (2007) *The Heart of Whiteness: Normal Sexuality and Race in America, 1880–1940*; Rosemarie Garland-Thomson’s (1996) *Extraordinary Bodies: Figuring Physical Disability in American Culture and Literature*; Lennard Davis’ (1995) *Enforcing Normalcy: Disability, Deafness, and the Body*; Robert McRuer’s (2006) *Crip Theory: Cultural Signs of Queerness and Disability*; Anna Creadick’s (2010) *Perfectly Average: The Pursuit of Normality in Postwar America*; and Ernst Walraud’s (2004) edited collection, *Histories of the Normal and the Abnormal: Social and Cultural Histories of Norms and Normativity*.

4. Examples of such reportage include Neil McMahon’s (2019) ‘The Year When Madness Became The New Normal’ (*Sydney Morning Herald*, 29 December) and Lyndon Schneiders’ (2020) ‘In This New Normal For Bushfires, Business As Usual Will No Longer Cut It’ (*The Australian*, 11 January).

5. See, for instance, Caroline Kormann’s (2020); or Will Peischel’s (2020).
